# ﻿*Petrocodonliboensis* (Gesneriaceae), a new species from Guizhou, China

**DOI:** 10.3897/phytokeys.243.125716

**Published:** 2024-06-12

**Authors:** Sheng-Hu Tang, Ming-Zhu Ou, Qi-Fei Ren, Jia-Wen Yang

**Affiliations:** 1 Guizhou Botanical Garden, Guiyang 550000, China Guizhou Botanical Garden Guiyang China; 2 National Forestry and Grassland Administration Key Laboratory for Biodiversity Conservation in Karst Terrain of Southwestern China, Guizhou Botanical Garden, Guiyang 550000, China National Forestry and Grassland Administration Key Laboratory for Biodiversity Conservation in Karst Terrain of Southwestern China, Guizhou Botanical Garden Guiyang China; 3 Gesneriad Conservation Center of China (Guizhou), Guizhou Botanical Garden, Guiyang 550000, China Gesneriad Conservation Center of China (Guizhou), Guizhou Botanical Garden Guiyang China

**Keywords:** Didymocarpoideae, flora of China, Guizhou, karst flora, new taxon

## Abstract

*Petrocodonliboensis* Sheng H.Tang & Jia W.Yang is a new species of Gesneriaceae from Guizhou, southwestern China. The new taxon has a pale-yellow corolla and is most similar to *P.luteoflorus*. However, it differs from the latter by having a urceolate (vs. cannulate) corolla tube, an abaxial corolla lip 0.8–1.1 mm (vs. 2–2.2 mm) long, and filaments 1.5–1.7 mm (vs. ca. 7 mm) long that are straight (vs. S-shaped or geniculate near the middle). The new taxon is assessed as “Data Deficient” (DD) according to the IUCN standards.

## ﻿Introduction

The genus *Petrocodon* Hance belongs to the family Gesneriaceae and subfamily Didymocarpoideae (Weber et al. 2013). *Petrocodon* was established in 1883 with a single species, *P.dealbatus* Hance (Hance 1883). Molecular studies have recently redefined the genus (Möller et al. 2011; Wang et al. 2011; Weber et al. 2011), which currently comprises 50 species and one variety (GRC 2024; IPNI 2024). This genus mainly occurs in China (POWO 2024).

During a field survey conducted in early July 2020 in Libo County, Guizhou Province, China, a population belonging to the genus *Petrocodon* (Gesneriaceae) was identified. Live plants with flower buds were cultivated in the Guizhou Botanical Garden. In late July 2020, they bloomed with pale-yellow flowers and for the first time, a corolla with straight filaments was observed. As the newly published species, viz., *P.luteoflorus* Lei Cai & F. Wen (Fan et al. 2020), which also has pale-yellow corollas, occurs in Libo County too, they were misidentified as *P.luteoflorus*. In late July 2021, they bloomed again, but only one picture of a flowering plant was captured by us because of the misidentification. In August 2022, the plants bloomed for the third time, and straight filaments were observed again. In September 2022, a field survey was conducted in Libo County to collect wild type plant specimens. The flowers of the cultivated and wild plants were identical.

In April 2017, live plants of *P.luteoflorus* (published in 2020) were introduced from Limingguan town, Libo County, which is its type locality, and bloomed in 2017 and 2018. In 2019, they perished due to inadequate care. Fortunately, we had already meticulously photographed the blossoms in 2017. In September 2022, we photographed the flowers at their original location with great care. The flowers of both cultivated and wild plants were indistinguishable. In November 2022, a second population without flowers was found in Yongkang Town, Libo County, and the shape and size of the leaf blades were the same as those of the plants from its type locality. Therefore, the leaf blade characteristics were stable.

The misidentified plants have stable characteristics different from *P.luteoflorus*, e.g., urceolate (vs. cannulate) corolla tube, abaxial corolla lip 0.8–1.1 mm (vs. 2–2.2 mm) long, and filaments straight (vs. S-shaped or geniculate near middle). Therefore, we conclude that the plants represent a new species.

## ﻿Materials and methods

One corolla was observed in 2020, and approximately 15 corollas of cultivated plants and 30 corollas of wild plants were observed in 2022. All observed corollas were pale-yellow and contained urceolate corolla tubes and straight filaments. Ten flowers in the field and ten flowers of cultivated plants were measured. A microscope (Olympus SZ61; Tokyo, Japan) was used for microscopic observations. The plants were described according to the terminology used by Wang et al. (1998). Relevant literature (e.g., Wei 2007; Jiang et al. 2011; Li and Wang 2015; Cen et al. 2017; Liu et al. 2020), especially those of the 12 accepted taxa of *Petrocodon* recorded in Guizhou, China (Wen et al. 2012; Guo et al. 2016; Li et al. 2019; Zhang et al. 2019; Fan et al. 2020; Xin et al. 2020; Pan et al. 2021; Zhang et al. 2023) was consulted. Specimen images of *Petrocodon* available in virtual herbaria and databases, including E (https://data.rbge.org.uk/search/herbarium/), K (http://apps.kew.org/herbcat/navigator.do), P (https://science.mnhn.fr/all/search), iPlant (containing specimen images of most herbaria in China, e.g., PE, IBSC, IBK, KUN) (http://www.iplant.cn/), and Global Plants (https://plants.jstor.org/) were examined. Specimens stored in CSH and the Guizhou Botanical Garden were also consulted.

## ﻿Results

### ﻿Taxonomic treatment

#### ﻿ Petrocodon liboensis

Taxon classificationPlantaeLamialesGesneriaceae

Sheng H.Tang & Jia W.Yang sp. nov.

7B2F579D-A38B-5390-9D68-655AD9779456

urn:lsid:ipni.org:names:77343416-1

Fig. 1

##### Diagnosis.

The new taxon has a nearly actinomorphic corolla and one nearly globose stigma. This combination of characters is shared by eight species and one variety of *Petrocodon*. The new taxon closely resembles *P.luteoflorus* (Fig. 2) in its pale-yellow corolla, two stamens, and divaricate thecae. However, it can be easily distinguished from the latter by the leaf blade being lanceolate or oblong (vs. narrowly elliptic or oblanceolate), lateral veins 2–3 (vs. 4–6) on each side of midrib, calyx segments 2.1–3.7 × 0.5–0.6 mm (vs. 6–8 × 1.5–2 mm) with entire (vs. entire to denticulate) margin, urceolate (vs. cannulate) corolla tube, abaxial corolla lip 0.8–1.1 mm (vs. 2–2.2 mm) long, and filaments 1.5–1.7 mm (vs. ca. 7 mm) long and straight (vs. S-shaped or geniculate near middle).

**Figure 1.**
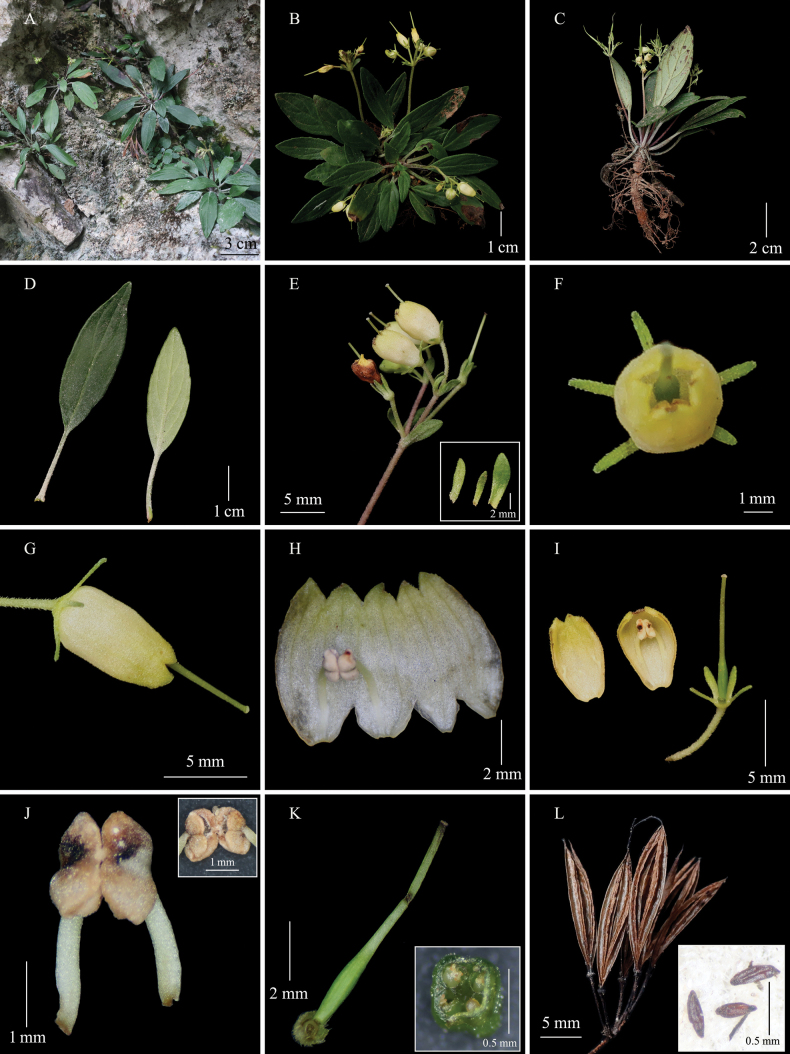
*Petrocodonliboensis***A** habitat **B, C** flowering plant **D** adaxial and abaxial surfaces of leaf blade **E** cymes, and bracts (inset) **F** flower in front view **G** flower in side view **H, I** opened corolla **J** stamens, and anthers (inset) **K** pistil, and cross section of ovary (inset) **L** capsules, and seeds (inset) (Photographs by Sheng-Hu Tang).

**Figure 2.**
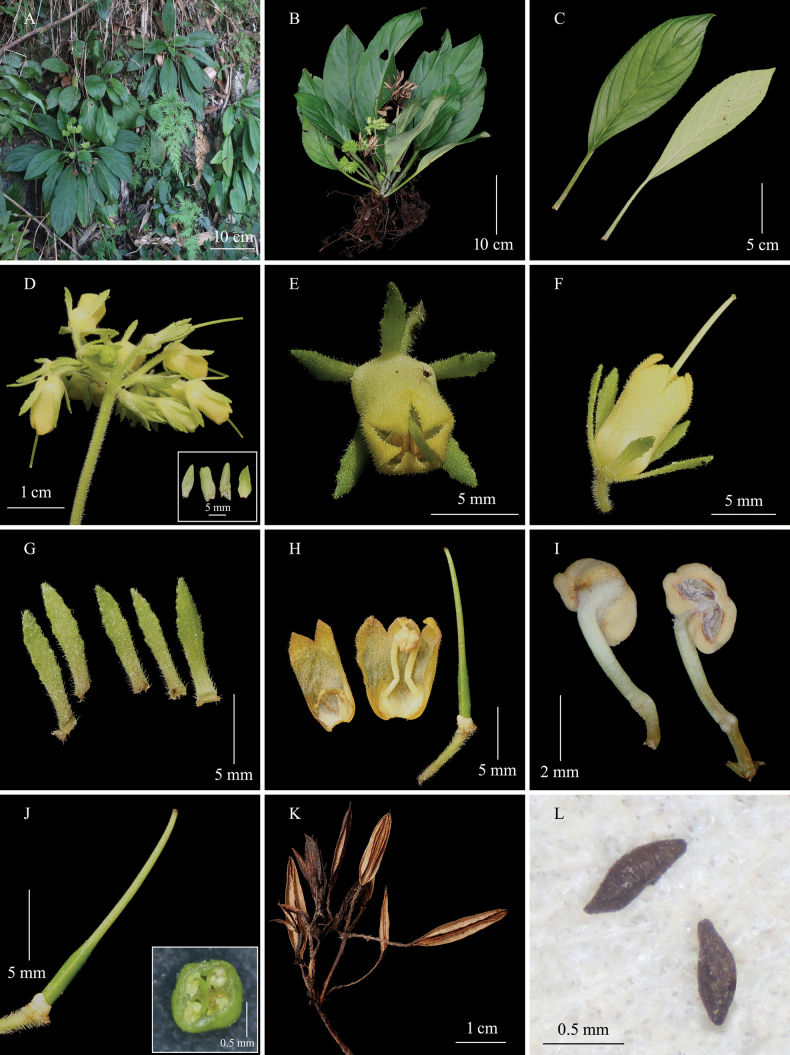
*Petrocodonluteoflorus***A** habitat **B** flowering plant **C** adaxial and abaxial surfaces of leaf blade **D** cymes, and bracts (inset) **E** flower in front view **F** flower in side view **G** calyx segments **H** opened corolla **I** stamens **J** pistil, and cross section of ovary (inset) **K** capsules **L** seeds (Photographs by Sheng-Hu Tang).

##### Type.

China. Guizhou Province: Libo County, Daqikong Scenic Spot, 25°16'N, 107°45'E, ca. 450 m elev., September 1, 2022, *Sheng-Hu Tang 202209001* (holotype: CSH! CSH0192992; isotype: the Guizhou Botanical Garden!)

##### Description.

Herbs, perennial, stemless. ***Rhizome*** terete, 5–55 mm long, 3.9–6.5 mm in diameter. ***Leaves*** 8–30, basal or crowded at rhizome apex; petiole 8.2–32 mm long, 0.9–1.8 mm in diameter, short strigose; leaf blade lanceolate or oblong, 26.4–82 × 11.3–19 mm, chartaceous, adaxially sparsely short strigose, abaxially densely short strigose along veins, base cuneate or broadly cuneate, margin entire to dentate or slightly repand, apex acute; lateral veins 2–3 on each side of midrib, adaxially inconspicuous or slightly impressed, abaxially slightly prominent. ***Cymes*** 1–4, axillary, 2–7 flowers per cyme; peduncle 35.8–59.2 mm long, 0.9–1 mm in diameter, densely short strigose; bracts 3, free, margin slightly crenulate, outside short strigose, inside sparsely short strigose, central one linear-lanceolate, 4.2–4.5 × 0.6–0.8 mm, lateral ones oblanceolate, 5.9–7.3 × 1.3–2 mm; bracteoles 3, same indumentum and shape as bracts, and slightly smaller than bracts in size, 2.5–3 × 0.9–1.2 mm. Pedicel 2.8–9.3 mm long, 0.3–0.4 mm in diameter, densely short strigose. ***Calyx*** nearly actinomorphic, 5-parted from base; segments equal or subequal, 2.1–3.7 × 0.5–0.6 mm, linear, both outside and inside short strigose, margin entire. ***Corolla*** pale-yellow, 5.3–8 mm long, outside puberulent, inside glabrous, 5-parted to 1/5, segments slightly converged; tube urceolate, 4.5–6.9 mm long, 2.1–3 mm in diameter at base, 1.7–2.1 mm in diameter at mouth; limb indistinctly 2-lipped, adaxial lip 2-parted from base, segments triangular, equal, 0.5–0.8 × 0.9–1.2 mm, abaxial lip 3-parted from base, segments triangular, subequal, 0.8–1.1 × 1.5–1.7 mm. ***Stamens*** 2, adnate to abaxial side of corolla tube 2.8–3.1 mm above base, included; filaments 1.5–1.7 mm long, ca. 0.2 mm in diameter, straight, glabrous; anthers dorsifixed, coherent at apex, elliptic, 1.3–1.7 × 0.8–0.9 mm, glabrous; thecae divaricate, confluent at apex, dehiscing longitudinally; connective not projecting; staminodes 3, central one inconspicuous, adnate to adaxial side of corolla tube 1.6–2 mm above base, ca. 0.1 mm long, lateral ones conspicuous, adnate to adaxial side of corolla tube 2.3–2.5 mm above base, 0.7–0.8 mm long. ***Disc*** ring-like, pale yellow, 0.2–0.3 mm high, margin entire or repand. ***Pistil*** 7.9–12 mm long; ovary linear, 2.8–4.5 mm long, 0.5–0.6 mm in diameter, glabrous or short strigose, 1-loculed, placentas 2, parietal, projecting inward, 2-cleft; style 5.1–7.5 mm long, ca. 0.3 mm in diameter, glabrous or sparsely short strigose; stigma 1, terminal, nearly globose, undivided. ***Capsule*** straight, linear, 10.3–17.8 mm long, 0.8–0.9 mm in diameter, dehiscing loculicidally to base; valves 2, straight, not twisted. ***Seeds*** unappendaged, fusiform, 0.4–0.6 mm long.

##### Phenology.

Flowering occurs from late July to early September, and fruiting in the wild is unknown; only capsules from the previous year were observed.

##### Etymology.

The new taxon was named after its locality in Libo County, China.

##### Vernacular name.

The Chinese name is “Lì Bō Shí Shān Jù Tái” (荔波石山苣苔).

##### Distribution and habitat.

Only one population was found at the Daqikong Scenic Spot, Libo County, Guizhou Province, China. Plants were found growing on wet shady rocks in the valley, along with mosses, weeds, and shrubs.

##### Preliminary conservation assessment.

Only one population of approximately 200 mature individuals was found in the type locality. It is highly likely that more populations were present in this area. Until further investigation, the species should be designated as “Data Deficient” (DD) according to the IUCN standards (IUCN 2022).

##### Taxonomic notes.

The new taxon is similar to eight species and one variety of *Petrocodon* in nearly actinomorphic flowers and one stigma. Its corolla tube is urceolate, similar to *P.scopulorum* (Chun) Yin Z. Wang (Chun 1946; Wang et al. 2011) and *P.urceolatus* F. Wen, H. F. Cen & L. F. Fu (Cen et al. 2017). The new taxon differs from *P.scopulorum* in its pale-yellow (vs. white) corolla, corolla parted to 1/5 (vs. 1/3), stamens 2 (vs. 4), and anthers coherent (vs. free). The new taxon is different from *P.urceolatus* by bracts 3 (vs. 2), pale-yellow (vs. white) corolla, puberulent (vs. glabrous) corolla outside, slightly constricted (vs. constricted) corolla mouth, and 1.7–2.1 mm (vs. ca. 0.9 mm) in diameter at corolla mouth. The new taxon was most similar to *P.luteoflorus* in terms of corolla color. The detailed morphological comparisons between *P.liboensis and P.luteoflorus* are presented in Table 1.

**Table 1.** Detailed comparisons between *Petrocodonliboensis* and *P.luteoflorus*.

Character	*P.liboensis*	*P.luteoflorus*
Leaf blade	lanceolate or oblong	narrowly elliptic or oblanceolate
Lateral veins	2–3 on each side of midrib	4–6 on each side of midrib
Calyx segments size	2.1–3.7 × 0.5–0.6 mm	6–8 × 1.5–2 mm
Calyx segments margin	entire	entire to denticulate
Corolla tube shape	urceolate	cannulate
Abaxial corolla lip length	0.8–1.1 mm	2–2.2 mm
Filaments	1.5–1.7 mm long and straight	ca. 7 mm long and S-shaped or geniculate near middle

## Supplementary Material

XML Treatment for Petrocodon liboensis

## ﻿Additional information

### Author ORCIDs

Sheng-Hu Tang https://orcid.org/0000-0002-2168-8559

Ming-Zhu Ou https://orcid.org/0000-0003-3417-0094

Qi-Fei Ren https://orcid.org/0000-0003-3265-5952

Jia-Wen Yang https://orcid.org/0000-0003-3427-714X

### Data availability

All of the data that support the findings of this study are available in the main text.
